# Pathways Linking Acculturation Profiles to Health Trajectories of Married Immigrant Women in South Korea

**DOI:** 10.3390/ijerph21111416

**Published:** 2024-10-25

**Authors:** Jihyoung Kim

**Affiliations:** Department of Child Welfare, College of Human Ecology, Chungbuk National University, Cheongju 28644, Republic of Korea; jkim2024@cbnu.ac.kr

**Keywords:** acculturation, health, married immigrant women, self-esteem, social network

## Abstract

This study investigated the health trajectories of married immigrant women in South Korea and examined the mediating role of psychosocial resources in linking acculturation profiles to health trajectories. A sample of 1635 mothers who participated in the Multicultural Adolescents Panel Study was examined. The results identified three distinct health trajectories, that is, high-stable, decrease-from-high, and low-stable. In addition, three acculturation profiles (maladaptive, Korean-oriented bicultural, and undifferentiated) indirectly predicted health class through self-esteem and social network. Specifically, self-esteem mediated the path from the maladaptive and Korean-oriented bicultural profiles to a stable-low health class. Further, social networks mediated the effect of the Korean-oriented bicultural profile on the decrease from the high health class. These findings highlight the heterogeneity of married immigrant women’s health and suggest the importance of considering the relationship between individual acculturation profiles and psychosocial resources to promote the health of married immigrant women.

## 1. Introduction

International migration is often linked to family formation, and this association is more pronounced among female migrants. South Korea, historically known as a culturally homogenous society, has seen a steady increase of multicultural families from 299,241 in 2015 to 399,396 in 2022 [1]. It is noteworthy that amid a decrease in the overall number of marriages, multicultural marriages in 2022 increased by 25.1% compared to the previous year [2]. Of these, 66.8% were married to Korean men and immigrant women [2]. The international marriage migration phenomenon in South Korea emerged from the combined demands of young women seeking economic opportunities from developing countries and old bachelors in rural areas marginalized in the domestic marriage market [3]. This unique background distinguishes married immigrant women from non-married immigrants. Specifically, they have a relatively short period from their initial meeting to marriage, usually promoted by international marriage-broker agencies [4]. Thus, they are more likely to experience marital conflicts associated with cultural and language differences [5,6]. Furthermore, most women experience childbirth within one year of marriage [7]. Considering that marriage and motherhood are challenging transitions, these events may act as a double burden and impair the health of married immigrant women. To date, most studies on the multicultural families in South Korea have focused on early adaptation, marital satisfaction, and childcare difficulties, and studies of immigrant women’s health rely on cross-sectional data and primarily focus on comparing their health status with that of women in the host country. However, some findings suggest heterogeneity in health status among immigrants. For instance, using eight major national data systems, including the National Vital Statistics System (NVSS) and the National Health Interview Survey (NHIS), Singh et al. [8] found varying health patterns across racial and ethnic groups. Similarly, a study of immigrant health in Genoa showed health inequalities in health-related quality of life, self-rated health, and morbidity within the immigrant population [9]. Despite this, relatively few longitudinal studies have examined individual variability in the health status of married immigrant women. Thus, using longitudinal data, this study aimed to investigate the heterogeneity of health trajectories among married immigrant women, specifically how their health status changes and whether distinct groups exist in this pattern of change. To achieve this, this study employed a latent class growth analysis (LCGA), a method effective for identifying different latent trajectory classes within a population. This individual-focused approach could inform the development of effective prevention and intervention strategies aimed at improving the health of married immigrant women.

Previous research has documented that individual acculturation types are associated with immigrants’ well-being and health [10,11]. Additionally, psychosocial factors, such as self-esteem and social networks, have consistently been identified as key determinants of immigrants’ health [12,13,14]. However, few studies have explored the roles of both acculturation and psychosocial factors in influencing subsequent health status. The current study hypothesizes that there are different groups of mothers with varying health trajectories based on individual acculturation types. Furthermore, psychosocial factors such as self-esteem and social networks are expected to mediate the relationship between the acculturation type and health trajectories. Given that the increase in the number of married immigrant women contributes to a growing segment of the population through childbirth, addressing the health status of this population and its precursors has implications for public health promotion.

## 2. Literature Review

### 2.1. Acculturation and Married Immigrant Women’s Health

Married immigrant women are usually socioeconomically vulnerable and experience health inequalities. Previous literature has documented that immigrants are more likely to be exposed to health risk factors such as language barriers, cultural differences, domestic conflicts, and social discrimination than the host population [15,16,17]. Furthermore, they navigate new cultures and learn about various systems, such as healthcare, education, and employment, often without the support of familiar social networks [18]. A review of 53 studies on international married women’s health in South Korea reported that immigrant women had more physical health problems, such as obstetrical and gynaecological health issues, obesity, and higher levels of depression and anxiety than Korean-born women [19]. Although considerable efforts in the immigration and health literature have been devoted to the health gap between the immigrant and host populations, there is a lack of empirical data on the developmental patterns of health status among married immigrant women in South Korea.

Individuals exposed to more than one culture must adapt to the cultural differences. Acculturation, which refers to cultural, psychological, and behavioural changes following sustained contact between two distinct cultures, has been suggested as an explanatory factor for the health disparities across and within immigrant populations. According to Berry’s bidimensional model, there are four different acculturation types across the axis of the acquisition of the new culture and the retention of the original culture: integration (maintaining the original culture and receiving the host culture), assimilation (identifying solely with the host culture), marginalization (rejecting both host and origin cultures), and separation (rejecting the host culture) [20]. The applicability of this bilinear model was supported by Yoon et al.’s meta-analysis, which showed that the association between acculturation and mental health was found only when using the bilinear measures of acculturation rather than the unilinear measures, which considered acculturation to be a linear process of adopting a new culture [21]. Furthermore, previous research has highlighted that the bilinear approach presumes the presence of all four types of acculturation, and provided evidence that multiple types of biculturalism could exist depending on the individual’s degree of receiving the host culture and maintaining the origin culture [22,23]. Accordingly, this study used a latent class analysis (LCA) to examine the distinct acculturation profiles of married immigrant women. An LCA allows for the identification of hidden or intermediate spaces in two-dimensional acculturation spaces by empirically extracting acculturation subtypes from a sample [24].

### 2.2. The Mediating Effects of Psychosocial Factors

Although acculturation and health may be directly linked, previous studies have suggested that psychosocial factors such as self-esteem and social networks mediate this association. For example, immigrant youths who pursued integration showed higher self-esteem than those who were marginalized, and youths who used assimilation or separation strategies showed intermediate levels [25]. Smith and Silva in their meta-analysis of 184 studies found that ethnic identity was more strongly related to self-esteem and positive well-being than to personal distress or mental health symptoms [26]. Regarding the association between acculturation and social support, which generally refers to perceived emotional and informational helping relationships, mainstream and ethnic acculturation were identified as significant predictors of social support. In addition, Paterson and Hakim-Larson found that when Arab migrants in Canada had a positive Arab culture orientation, they were more likely to have trusted family members, friends, and significant others with whom to share their concerns and needs, thereby contributing to their life satisfaction [27]. Similarly, the orientation to the host culture of Iraqi refugees in the United States was positively associated with social support, which, in turn, was negatively associated with depression and PTSD symptoms [28]. Although the structure and content of social networks change throughout life, the effects of social networks on health are prominent among immigrants who have experienced the loss of existing social networks and must find alternatives in a new country within a relatively short time [29].

Psychosocial resources are well-established as major health factors. Previous studies have shown that social ties positively influence health behaviours throughout life [30,31]. For example, individual perceptions of the availability of helpful relationships within social networks are especially beneficial, because they constitute individual-level resources that buffer against detrimental emotional and physiological reactions to stressful events [32]. In addition, self-esteem, control beliefs, and optimism have direct positive effects on the physical, functional, and subjective health in all SES groups [33]. Similarly, self-esteem buffers the negative impact of perceived discrimination on psychological distress in ethnic minority groups [34]. Therefore, this study hypothesized that self-esteem and social network as psychosocial resources mediate the relationship between acculturation profiles and health trajectories.

### 2.3. Current Study

Despite accumulating evidence of the health gap between immigrants and the host population, heterogeneity in changes in the health status among married immigrant women and its predictors have not been well investigated, particularly in the South Korean context. This study addresses this gap by examining distinct health trajectories in the sample. Furthermore, based on the prior literature indicating the multifaceted nature of acculturation, a latent class analysis was used to identify different acculturation profiles with similar patterns of scores for acculturation dimensions. These results would contribute to an understanding of the health disparities among married immigrant women. Additionally, by exploring the long-term pathways connecting acculturation to health, and identifying changeable mediating factors that can be addressed through interventions, this study provides specific recommendations for enhancing the health of married immigrant women.

## 3. Materials and Methods

### 3.1. Sample

The data for this study were obtained from the Multicultural Adolescents Panel Study (MAPS), a nationally representative longitudinal study of 1635 multicultural adolescents and their mothers in South Korea. The MAPS, conducted by the Korea Youth Policy Institute, recruited participants using a stratified sampling of multicultural students enrolled in the fourth grade in 16 cities throughout Korea. The majority of adolescents in the sample distribution are from families with international marriages. A total of 1635 fourth-grade students and their mothers participated in the first wave of data collection (2011) and will be followed until 2025. The MAPS data were collected via CAPI (Computer Assisted Personal Interviewing) with the assistance of trained professionals who visited the adolescents’ homes. The mothers were provided with a questionnaire translated into nine languages, along with the Korean questionnaire. Further information on the MAPS is provided at https://www.nypi.re.kr/archive (accessed on 24 September 2024). As this study aimed to investigate the health trajectories among married immigrant women, the participants who failed to respond to questions on health status and were Korean-born (*n* = 339) were excluded. Consequently, 1286 married immigrant women who participated in waves 4, 5, and 6 were included in the final sample. The sample comprised 33.7% Korean-Chinese, 22.6% Chinese, 17.7% Vietnamese, 6.0% Japanese, and 5.2% Filipinos.

### 3.2. Measures

#### 3.2.1. Health Status

The health status was measured using a self-reported rating ranging from 1 (poor) to 5 (excellent). To examine the health trajectories of the immigrant women, this study used data from waves 4, 5, and 6. Previous studies demonstrated that self-rated health (SRH) predicts the quality of life, disease morbidity, and mortality rate; thus, it can serve as a principal health indicator for monitoring individual health status [35,36].

#### 3.2.2. Acculturation Profiles

To identify the distinct profiles of acculturation, the East Asian Acculturation Measure (EAAM) assessed at wave 4 was used. The EAAM is a 29-item self-report inventory that measures Berry’s four dimensions of acculturation: assimilation, separation, integration, and marginalization [37]. The four dimensions were originally composed of eight, seven, five, and nine items rated on a scale of 0 (not at all) to 3 (very much). The MAPS used 26 items, excluding one item (i.e., I would prefer to go out on a date with an Asian than with an American) from the separation dimension, reflecting the contemporary situations and characteristics of East Asian people from China, Japan, and Korea. Cronbach’s α was 0.85 for assimilation, 0.74 for separation, 0.71 for integration, and 0.92 for marginalization.

#### 3.2.3. Psychosocial Resources: Self-Esteem, Social Network

Self-esteem was assessed using nine items from the Rosenberg Self-Esteem Scale [38]. The responses to each item ranged from 0 (not at all) to 4 (very much). The sample items include, ‘On the whole, I am satisfied with myself’ and ‘I feel that I’m a person of worth’. A sum score was computed, with higher scores reflecting positive self-esteem (α = 81). Social network, as a dichotomous variable, was assessed by asking participants whether they had supportive social relationships.

#### 3.2.4. Covariates

Several covariates related to acculturation and health were also included, including the length of residence [39], family income [40], age [41], language proficiency [42], and education level [43]. The mothers reported their length of residence in Korea, family income, age, educational attainment on a 5-point scale (<high school, high-school graduate, associate degree, bachelor’s degree, postgraduate degree), and Korean proficiency in speaking, writing, reading, and listening, which ranged from 4 to 20 points.

### 3.3. Data Analysis

A latent class growth analysis (LCGA) using Mplus Version 7.0 was used to identify qualitatively distinct patterns in health status. The optimal number of classes was determined by the highest log-likelihood (LL) values, smallest Akaike information criterion (AIC), Bayesian information criterion (BIC), entropy greater than 0.85, and a significant *p*-value in the Lo–Mendell–Rubin likelihood-ratio test (LMR-LRT) [44]. The size and interpretability of the classes were also considered [45]. Once the health trajectories were identified, multinomial regression was performed to examine the difference in the probability of class membership according to the covariates. A composite acculturation scale was developed using a latent class analysis to examine the distinct acculturation profiles among the samples. The hypothesized longitudinal pathways, including acculturation profiles, social network, and self-esteem, were tested using a path analysis. Finally, the indirect associations between the acculturation profiles and health status through self-esteem and social network were tested. The significance of the indirect effects was tested using MODEL CONSTRAINT in Mplus [46]. All analyses included demographic characteristics as control variables. Missing data were accounted for using a full-information maximum-likelihood procedure.

## 4. Results

### 4.1. Identification of Different Health Trajectory Classes

**Figure 1:**
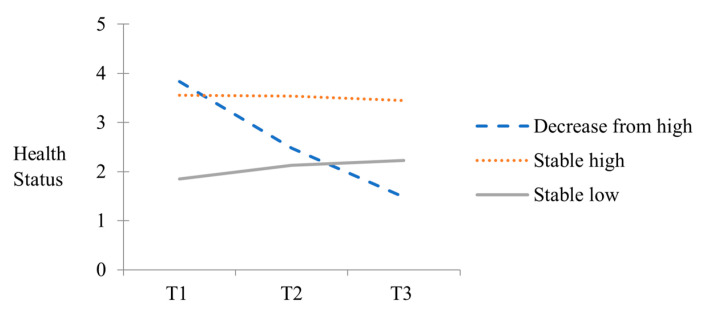
Latent trajectory classes of health status.

As shown in Table 1, both the AIC and BIC decreased and the entropy value increased consistently for the one-class to three-class models. Although the four-class model had lower AIC and BIC values than the three-class model, it did not show a significant *p*-value in the LMR test. Therefore, this study chose a three-trajectory class as the best solution to describe the underlying heterogeneity in the overall health status of the sample. Figure 1 illustrates the group-averaged health trajectories exhibited by each of the three identified classes.

As shown in Figure 1, the first class (*n* = 28, 2.1%), which initially exhibited the highest level of health, showed a steep decline throughout the study period (slope M = −1.001, SE = 0.264, *p* < 0.001) and was labelled as the decrease-from-high class. The second class (*n* = 1148, 89.3%) maintained, on average, a higher level of health than the other two classes, although there was a slight decline (slope M = −0.053, SE = 0.019, *p* < 0.01), and was labelled as the stable-high class. The third class (*n* = 110, 8.6%) showed consistently low levels of health and was labelled the stable-low class. This class showed a relatively higher level than the first class after T2; however, the changes in this class were not significant over the study period (slope M = 0.126, SE = 0.117, *p* < 0.281).

The probability of membership in a particular latent class was partly conditional on the demographic measures, as presented in Table 2. In general, the perceived family income and education were consistent predictors of a stable-high class membership. Specifically, women in the stable-high class reported higher levels of perceived family income and education than did those in the decrease-from-high and stable-low classes. In addition, women in the stable-low class were significantly older than those in the decrease-from-high and stable-high classes. There were no significant differences in the Korean-language proficiency or length of residence between the three classes.

### 4.2. Pathways Linking Acculturation Profiles to Health Trajectories

**Figure 2:**
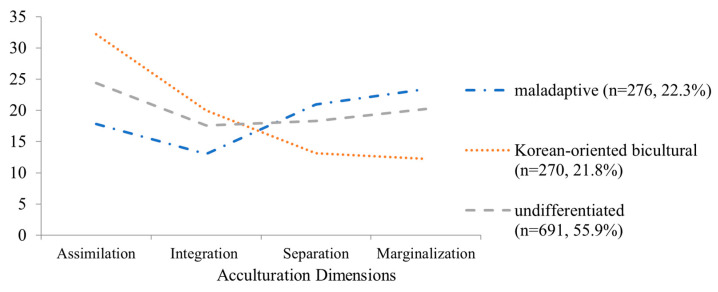
Acculturation profiles.

Prior to the path analyses, a latent class analysis utilising four dimensions of acculturation measures (i.e., assimilation, integration, separation, and marginalization) was conducted to identify the acculturation profiles. A three-class solution with a lower AIC and BIC best fit the data (AIC = 27,536.372, BIC = 27,720.708). In addition, the three-class model demonstrated fair entropy (0.804) and significant L-M-R values (*p* < 0.001). As shown in Figure 2, the first class (*n* = 276, 22.3%) was characterized by high levels of separation and marginalization, and low levels of assimilation and integration. Based on empirical evidence that marginalization and separation are associated with poorer health-related outcomes than integration and assimilation strategies [47,48], this group was labelled as the maladaptive profile. In contrast, the second class (*n* = 270, 21.8%) was characterized by low levels of separation and marginalization, and high levels of assimilation and integration. This group showed significantly lower scores for separation and marginalization and higher scores for assimilation and integration than the maladaptive profile. Since this group showed relatively higher levels of assimilation than integration, it was labelled the Korean-oriented bicultural profile. The third class (*n* = 691, 55.9%) maintained similar levels across four dimensions and did not show any preference for any particular acculturation type. Therefore, it was labelled as the undifferentiated profile. Analysis of variance and descriptive statistics of indicators of acculturation profiles were shown in Table 3. 

**Figure 3:**
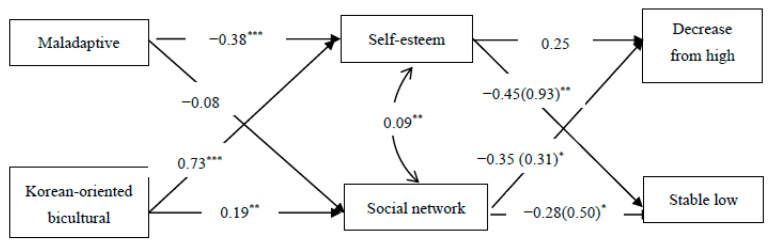
Model predicting pathways to health trajectory classes. * *p* < 0.05, ** *p* < 0.01, *** *p* < 0.001.

Figure 3 shows the results of the mediational process linking the acculturation profiles to health status through psychosocial resources. As expected, acculturation profiles were associated with psychosocial factors. Specifically, immigrant women with the maladaptive profile showed lower self-esteem than those with the undifferentiated profile (β = −0.38, *p* < 0.001). Also, the Korean-oriented bicultural profile was associated with higher self-esteem (β = 3.44, *p* < 0.001) and higher levels of social network (β = 0.73, *p* < 0.001) than the undifferentiated profile. The psychosocial factors, in turn, significantly predicted the health classes. Specifically, high self-esteem was associated with decreased odds of being included in the stable-low health class compared to the stable-high health class (RRR = 0.93, *p* < 0.01). High levels of social networks were also associated with decreased odds of being included in both decrease-from-high and stable-low health classes (RRR = 0.31, *p* < 0.05; RRR = 0.50, *p* < 0.05, respectively). An indirect-effect analysis revealed that self-esteem significantly mediated the path from the maladaptive profile to the stable-low health class (b = 0.14, *p* < 0.05). The effect of the Korean-oriented bicultural profile was indirectly associated with the stable-low health class through self-esteem (b = −0.27, *p* < 0.05), and marginally predicted the decrease-from-high health through a social network (b = −0.04, *p* < 0.08).

The relative risk ratios (RRR) of the health trajectory classes are shown in parentheses. The coefficients for the maladaptive and bicultural profiles were relative to the undifferentiated profile. The reference for health status was a stable-high health class. The respondents’ perceived family income, education level, and age were controlled. The direct paths from the acculturation profiles to health classes were not significant, and are not shown for clarity.

## 5. Discussion

This study aimed to examine the distinct health trajectories of married immigrant women and the mediating roles of psychosocial resources in linking acculturation profiles to health trajectories. Consistent with the hypothesis, the results identified three trajectory health classes, each with its own level and process of change. The majority of the sample (89.3%) maintained healthy status throughout the study period. Diverse government- and community-initiated services have been implemented in South Korea in response to the rapid increase in the number of married immigrants. Since the Multicultural Family Support Act was enacted in 2008, multicultural family support centres across the country have provided nutrition and health education, as well as medical services, such as the dispatch of antenatal and postpartum helpers and health check-ups. These factors may have contributed to the health of married immigrant women. The other two groups, including the decrease-from-high (2.1%) and stable-low (8.6%) groups, exhibited an overall unhealthy status compared with the stable-high group, and low levels of family income and education increased the probability of being in these two groups relative to the stable-high group. These findings are consistent with previous evidence that socioeconomic factors play critical roles in health disparities [49]. The current results highlight the importance of targeted programmes and policies for married immigrant women, particularly those from disadvantaged backgrounds.

Before the path analyses, a latent class analysis was performed using four dimensions of acculturation measures (i.e., assimilation, integration, separation, and marginalization) to identify the acculturation profiles in the sample. The results show that a three-profile solution fit the current sample. Specifically, half of the participants (*n* = 691, 55.9%) comprised the undifferentiated profile and did not show any preference for a particular type of acculturation. Additionally, they showed similar levels in all four acculturation dimensions. The maladaptive profile (*n* = 276, 22.3%) showed high levels of separation and marginalization and low levels of assimilation and integration. Conversely, the Korean-oriented bicultural profile (*n* = 276, 22.3%) was high in assimilation and integration and low in separation and marginalization. The existence of the undifferentiated and Korean-oriented bicultural profiles is consistent with the findings of Schwartz and Zamboanga’s study [24]. Using a latent class analysis, they identified six distinct acculturation classes among Hispanic college students: assimilated, partially bicultural, separated, fully bicultural, undifferentiated, and American-oriented. In their study, the undifferentiated class endorsed all four acculturation dimensions, which is similar to the current analysis. Notably, their undifferentiated class showed the lowest levels on nearly all indices of cultural identity compared to the other five classes; thus, these individuals were described as marginalized and somewhat confused about their cultural identities. However, the undifferentiated profile in the current study showed intermediate scores for all four acculturation dimensions. Thus, it might be more reasonable to posit that they are in the process of finding their own cultural identities by navigating both heritage and new cultures, rather than losing all cultural affiliations and developing de-identified cultural identities.

This study also found a Korean-oriented bicultural profile, characterized by mixing biculturalism with assimilation. This finding is similar to that of Schwartz and Zamboanga’s (2008) findings, who identified two subtypes of integration: American-oriented biculturalism and partial biculturalism [24]. This also supports the assertion that acculturation categories may not be independent. Also, the existence of the maladaptive profile, with the combination of marginalization and separation, supports the previous study that failed to find Berry’s ‘pure’ marginalization group [50]. Based on empirical evidence of low or non-existent marginalization groups, researchers questioned the validity of the marginalization concept and noted that it is implausible for someone to develop a cultural identity that does not incorporate either heritage or receiving cultural contexts [50,51].

Overall, these findings have methodological and theoretical implications. First, using a person-centred approach to identify the acculturation profiles, the current study adds evidence to prior research showing the applicability of LPA in analysing acculturation types. The existence of distinct acculturation profiles suggests that the preference for one strategy over others is not a zero-sum game and immigrant women may combine several strategies to effectively adapt to the new environment. Previous research has highlighted that adaptation to mainstream society can occur selectively and disparately depending on context and location, such as more cultural maintenance in private spheres or domains, such as the home and ethnic community; and more acquisition of a new culture in public spheres, such as the workplace or politics [20]. Similarly, an empirical study on parenting and acculturation found that immigrant parents experience a negotiating process regarding which cognitions or practices to retain from their indigenous culture, which to modify, and which new conventions to adopt [18]. Future research could provide a more detailed description of the acculturation process using a time diary or in-depth interviews.

The results of the current study elucidated the psychosocial mechanisms that link acculturation profiles to health trajectory classes, suggesting that the consequences of acculturation are highly variable depending on the social and personal variables. Specifically, the Korean-oriented bicultural profile showed higher self-esteem and social network levels than the undifferentiated profile did. In addition, the maladaptive profile showed lower self-esteem than the undifferentiated profile. This supports previous findings that mainstream and ethnic acculturation are important predictors of social support, which contributes to health [27,28,51]. According to the bioecological model of human development, the cultural transitions that accompany immigration can cause complex disruptions in both proximal and distal environments. Under normal conditions, the proximal processes function as buffers against the adverse effects of macrosystem changes on human development. However, changes in the cultural context usually exceed an individual’s capacity to cope with them because of their magnitude and speed, which, in turn, leads to serious psychological distress [52]. Consequently, the imbalances between environmental demands and personal resources consistently require immigrants to make efforts to restore their sense of loss of control.

The finding that the women with Korean-oriented bicultural profiles had more social networks and higher levels of self-esteem indicates that they successfully compensated for the loss of psychosocial resources by affiliating themselves with a new mainstream and ethnic culture. In contrast, the immigrant women with maladaptive profiles, characterized by high levels of marginalization and separation, failed to secure psychosocial resources, leading to a stable-low health status. These findings are consistent with those of previous studies indicating that individuals who become integrated or have bicultural experiences have less acculturative stress [53] and psychological problems than those who choose to marginalize or separate [22]. Furthermore, given that the Korean-oriented bicultural profile represents a combination of assimilated and integrated categories, it can be inferred that inhabiting residential culture could make it easier to find health-related psychosocial resources. This result is consistent with previous findings that identification with a dominant society, as observed in integration and assimilation, has been linked to less distress, less depressive symptomology, and increased social adjustment [54]. Similarly, blended biculturalism has been associated with favourable psychosocial functioning, such as self-esteem and depression [55].

As expected, psychosocial characteristics were significantly associated with health trajectories, suggesting a mediating pathway linking acculturation profiles to health trajectories. The results clearly showed that higher self-esteem and social network levels were associated with a stable-high health class. Mounting evidence indicates that psychological and social factors such as self-esteem, control beliefs, and perceived emotional and informational support are resources for functional and subjective health [32,56]. Regarding the beneficial effects of self-esteem on health outcomes, previous research has suggested that self-esteem acts as a buffer against external stressors [57]. Consistent with this, the current study found that lower self-esteem increased the odds of being included in a stable-low health class compared with a stable-high health class. The finding that the stable-low health class showed lower self-esteem than the decrease-from-high health class further suggests that self-esteem involves the maintenance of health status rather than its change.

Individual perceptions of the available social networks are also important for health. Specifically, social networks were associated with being in the stable-high health class. Previous studies have consistently reported that social networks directly influence health by providing material aid, services, and health-related information, or indirectly by buffering the emotional and physiological reactions from stressful external events by strengthening the sense of intimacy, attachment, and reassurance [30,31]. For example, Park et al. [58] found an association between less frequent contact with intimate relationships and lower health levels in a Korean sample. Married migrant women usually experience acculturative stress, loneliness, and family conflicts associated with cultural and language differences, which are detrimental to their mental health. Relationships with various social groups have been suggested as major channels through which social, emotional, and instrumental support is exchanged, thereby mitigating the adverse effects of immigration [59,60].

This study has several limitations. Acculturation is an interactive process between migrants and the sociocultural context in which they settle, rather than being a property of the migrants themselves [61]. In other words, the integration of immigrants is likely if the host society protects sociocultural diversity and ensures that all individuals have equal access to opportunities without discrimination and exclusion based on race, ethnicity, and nationality. In addition, if the host society prefers assimilation into the host culture, and immigrants’ acculturation mode is not consonant with expectations, their health status might deteriorate. Future research should provide more detailed information on acculturation by incorporating additional macro factors such as the host country’s socio-political climate, public and media awareness of minority groups, and neighbourhood characteristics. In addition, this study focuses on married immigrant women in South Korea. Because every migrant group in a country has a unique migration history, the results may not be generalizable to other migrant groups. Considering that acculturation is a multifaceted feature encompassing changes in values and behaviours [62,63], follow-up research examining acculturation measured by multiple assessments may elucidate whether the discovered profiles are measure-specific or generalizable. Due to data limitations, this study uses only Berry’s acculturation scale to uncover acculturation profiles. Even though the three distinct profiles were validated through their relationships with psychosocial outcomes, future studies should strengthen the external construct validity by examining the differential association between acculturation profiles and other culture-related correlates, such as ethnic identity, ethnic socialization, and perceived ethnic discrimination.

Another limitation relates to the way in which health status was assessed. This study used the immigrant women’s self-reports of their health. To reduce concerns about potential misreporting bias, future studies might consider more objective measures of health.

## 6. Conclusions

Previous studies have reported on the effects of acculturation on immigrant health. This study advances the literature by identifying the heterogeneity in health status among immigrant women and describing how acculturation profiles mediated by psychosocial resources are associated with health trajectories. These findings suggest that adopting a ‘one-size-fits-all’ approach may be ineffective and underscore the importance of acknowledging immigrants as a diverse population. Moreover, by investigating the mediating roles of psychosocial factors in the link between acculturation and health status, this study suggests that establishing community networks, self-help groups, and providing counselling services to enhance self-esteem would promote health equity among immigrant women. In addition, the finding that the married immigrant women with maladaptive acculturation profiles were more likely to have low self-esteem and thus were at a greater risk of poor health status suggests that their health issues can be prevented or reduced by targeted intervention programmes that improve self-esteem.

## Figures and Tables

**Table 1:** Identification trajectory classes.

Classes	AIC	BIC	SSA-BIC	Entropy	LMR *p*
One	8637.861	8669.201	8650.141	-	-
Two	8596.579	8648.608	8621.311	0.766	0.0149
Three	8575.482	8648.608	8597.574	0.828	0.0043
Four	8548.868	8637.664	8583.662	0.803	0.0056

AIC = Akaike information criterion; BIC = Bayesian information criterion; SSA-BIC = sample-size adjusted Bayesian information criterion; LMR = Lo-Mendell-Rubin likelihood ratio test.

**Table 2:** Demographic covariates per trajectory measured by mean and standard deviation.

Demographics	Decrease from High (*n* = 28, 2.1%)	Stable High (*n* = 1148, 89.3%)	Stable Low (*n* = 110, 8.6%)
	M(SD)	M(SD)	M(SD)
Perceived family income	1.87 (0.82)	2.37 ** ^(^***^)^ (0.73)	1.95 (0.75)
Education level	2.13 (1.06)	2.51 * ^(^**^)^ (0.90)	2.22 (0.86)
Age	42.09 (5.02)	43.44 ^(^*^)^ (5.13)	44.79 * (5.56)
Korean language ability	12.41 (2.52)	12.33 (2.17)	12.03 (2.26)
Length of residence (months)	181.68 (33.83)	185.16 (38.25)	182.47 (53.17)

* *p* < 0.05, ** *p* < 0.01 with reference to the decrease-from-high class; ^(^**^)^ *p* < 0.01, ^(^***^)^ *p* < 0.001 with reference to the stable low class.

**Table 3:** Analysis of variance and descriptive statistics of indicators of acculturation profiles.

Indicators	Acculturation Profiles			*F*
	Maladaptive M(SD)	Korean-Oriented Bicultural M(SD)	Undifferentiated M(SD)	
Assimilation	17.80 (3.04)	32.15 (3.94) ^a^***^, b^***	24.34 (3.81) ^a^***	620.169 ***
Integration	13.03 (2.15)	19.98 (2.66) ^a^***^, b^***	17.58 (2.01) ^a^***	481.872 ***
Separation	20.95 (3.65)	13.13 (3.69) ^a^***^, b^***	18.31 (2.98) ^a^***	269.516 ***
Marginalization	23.43 (5.75)	12.21 (4.12) ^a^***^, b^***	20.22 (5.93) ^a^***	180.910 ***

(*N* = 1237); a: reference is maladaptive profile, b: reference is undifferentiated profile; *** *p* < 0.001.

## Data Availability

Publicly available datasets were analyzed in this study. The data of the Multicultural Adolescents Panel Study (MAPS) can be found here: https://www.nypi.re.kr/archive/mps (accessed on 24 September 2024). The data generated in this study are available on request from the corresponding author. The data are not publicly available due to privacy.
